# Construction of a Traceability System for Food Industry Chain Safety Information Based on Internet of Things Technology

**DOI:** 10.3389/fpubh.2022.857039

**Published:** 2022-05-27

**Authors:** Siyuan Lin, Qi Shi, Ning Zhou

**Affiliations:** ^1^Institute of Food and Strategic Reserves, Nanjing University of Finance and Economics, Nanjing, China; ^2^School of Economics, Nanjing University of Finance and Economics, Nanjing, China

**Keywords:** Internet of Things technology, food industry chain, traceability system, industry chain, safety system

## Abstract

The continuous development of the social economy, has stimulataed an increase in the satndard of living and increased the deman for consumption resulting in the demand for high-quality and safe food has continued to increase. The so-called food safety means that the food that people eat under certain conditions will not harm human health. Frequent food safety incidents have highlighted the seriousness of my country's food safety problems and exposed loopholes in my country's food safety supervision. This article aims to study the construction of the Internet of Things technology in the food industry chain safety information traceability system, research on the RFID technology, GPS technology, and sensor technology in the Internet of things technology, and also conduct some research on the modules of the food industry chain safety information traceability system. This paper proposes to integrate the Internet of Things technology into the construction of the food industry chain safety information traceability system. First, a detailed analysis of some of the technologies that may be used is carried out, and then through the investigation of people on food safety and other aspects, and the food traceability system satisfaction survey. The experimental results in this article show that 40% of women pay more attention to food safety. Of course, in the satisfaction survey of the food safety traceability system based on the Internet of Things technology, it has been recognized by more than 20% of the people.

## Introduction

In recent years, worldwide food safety accidents have occurred frequently, leading to a wide range of serious consequences. Continued food safety incidents not only seriously threaten the health of consumers, but also affect the healthy, sustained and stable development of the food industry. Food safety issues have attracted more and more attention from consumers who are more and more aware of food safety, and the supervision of news organizations and public opinion has also played a certain role. On the other hand, there are still considerable loopholes in our food safety supervision system. Fake eggs have been a threat for more than ten years without supervision. The society needs the establishment of a higher-level food safety system. Food safety issues are not as simple as government supervision, but have risen to social, economic and people's livelihood issues. How food safety issues are handled is a manifestation of the government's concept of governance and other aspects, which in essence severely tests all levels of government. Therefore, in recent years, people have been keen to discuss how to effectively supervise food safety. The current problem that needs to be solved is how to effectively pre-warn food safety issues in the safety supervision of food circulation, and how to deal with emergencies in a timely and proper manner, so that normalized supervision can reach the level of efficiency, and the most timely and effective way to get these problems. The solution is to establish a food safety traceability system. The food quality chain involves multiple links, mainly including the quality control of raw materials and the transportation of raw materials at the place of production of raw materials, the control of product production processes by food processing plants, the control of product quality, and the storage of products by sales intermediaries, until use by end customers. If people want to eat healthy food, they must supervise all aspects of the food and control every aspect in order to ensure the quality and safety of the products.

The creation of a safety information traceability system for the food industry chain not only makes an important contribution to enhancing the overall image of the industry, building a good corporate brand, and enhancing the core competitiveness of the company. At the same time, the establishment of a food safety traceability system has increased the depth of supervision of the government's law enforcement and supervision departments, improved supervision efficiency, and established a set of standards and systems for a food safety traceability platform. From a technical perspective, the Internet of Things technology is used to solidify methodologies such as HE in the software and hardware products, and promoted and applied in the traditional food planting, breeding, processing, logistics, and circulation industries. Technological innovation in traditional industries plays a powerful role in promoting the mature and reliable use of RFID, GPS and other technologies. In the development of the food industry, the establishment of a public food safety traceability information query and service platform provides an expandable basic platform for the promotion of other traceability systems in the fast-moving consumer goods field. It has played a significant role in promoting the construction of traceability information service systems in other agricultural and sideline products, poultry, fruit and other industries. At the same time, the establishment of an information security traceability system for the food industry chain based on the Internet of Things technology ensures that consumers have the right to know all aspects of food production. The food safety traceability system establishes a standard identification system that meets international standards in all aspects of food logistics, and ensures that all aspects of the supply chain are properly identified, improving the reliability of product sources and the speed of information transmission and processing, and integrating electronic data Exchange, e-commerce and world trade are combined.

Internet of things technology is widely used. In an article, Zhao-Hui elaborated on the digital construction of museums and proposed the idea of using the Internet of Things technology to build digital museums. Intelligent navigation based on RFID technology provides a new way for the construction of intelligent museums (1). In coal mining, Dong et al. (2) conducted a research experiment on the safety and maintenance of coal mine plant systems as an example, which changed the current model of coal mine plant management and ensured the safe and highly reliable operation of coal mine plant. GPS, as a part of the perception level of the Internet of Things technology, is also being used more and more widely. Braun et al. presented a direct comparison of non-isotropic, integrated water vapor measurement between a ground-based global positioning system (GPS) receiver and a water vapor radiometer (WVR). Use the multi-path map of a specific site to perform station-related error correction on GPS observations (3). With the continuous development of social science and technology, food safety has attracted more and more attention. Scott pointed out in the study that the incidence of foodborne diseases is increasing globally. Although the food-borne disease data collection system often misses a large number of sporadic infections in the home, it is now generally accepted that many food-borne diseases are caused by consumers improperly handling and preparing food in their own kitchens (4). In the research on food safety issues, He et al. proposed various ARGs and antibiotics that are ubiquitous in pig manure and wastewater. Most ARGs and antibiotics survive in the farm waste treatment system for food safety and human health (5). Similarly, the Hsu et al. (6) study found that subjective understanding of organic food, health awareness and food safety concerns are important factors that affect the willingness to buy organic food. As food safety has attracted more and more attention, the food industry chain safety information traceability system has gradually entered the public's field of vision, and is gradually affecting people's lives. Hong established a research model in a study to analyze the impact of supply chain quality management and traceability systems on the quality performance and financial performance of companies in the food, pharmaceutical and automotive industries. And use the traceability system as a parameter to investigate the impact of the appropriate actions of the enterprise on the consumer's product quality complaints on the performance of the enterprise (7). Although the development of the Internet of Things technology and the information security traceability system of the food industry chain has promoted the improvement of food safety. However, these technologies and systems are still subject to restrictions and power, network restrictions in use, and the actual use costs are also relatively large.

The innovation of this article lies in the integration of modern Internet of Things technology into the traceability system of food safety. Through the use of related technologies of the Internet of Things, such as video surveillance technology to supervise the brotherhood of food production, people can connect through video. Line check the production situation of various environments such as food raw material production, food processing, and food transportation (8). For another example, GPS technology can be used to locate the food from where it was transported, where it has been, when it arrived, etc. to conduct a supervision to ensure that the food is standardized and reasonable throughout the transportation process, so as to ensure that food safety is not affected during the transportation process (9). By applying the Internet of Things technology to the food safety traceability system, it is not only an emphasis on food safety, but also a concern for the people's livelihood by the governing and law enforcement agencies. Improving people's food safety and protecting people's physical and mental health through modern scientific and technological means is the goal of scientific and technological development, and it is also what people are constantly looking forward to.

## Application Method of Internet of Things Technology in Food Safety Industry Chain Safety Information Traceability System

### Internet of Things Technology

Simply put, IoT technology is the connectivity of all objects to the Internet through radio spectrum recognition and other message detection devices for intelligent identification and administration (10). In other words, IoT is the new technology that connects various sensors to the available network. The Internet of Things is another technological revolution in the information industry, representing the future development trend of computers and communications, and affecting the future social and economic development (11). The Internet of Things is the fusion of some technologies active in various fields, and these technologies have already had some applications (12, 13). In the food industry chain information security traceability system, a large number of Internet of Things technologies must be used. This article studies and describes the more important technologies.

#### Rfid Technology

Radio spectrum recognition uses radio wave high speed message exchange and storage technology, combined with wireless communication and data acquisition technical, connected to the database system, to achieve non-contact two-way communication, so as to achieve the identification purpose of data exchange. And the complex system is connected in series (14).

**Figure 1:**
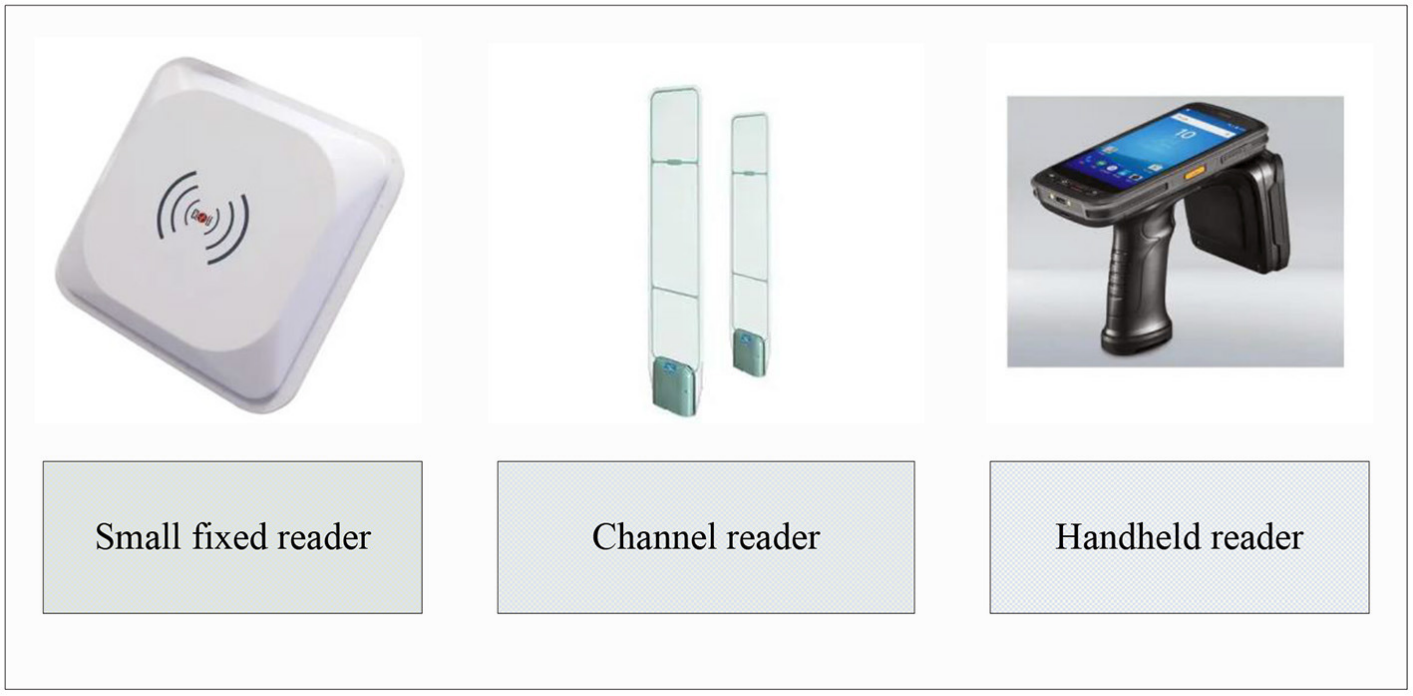
Commonly used readers.

Radio frequency identification technology uses the three components of tags, readers and computer systems to identify item information while transmitting information (15). Among them, the RFID tag is mainly used to store the product information of the identified object and has unique identification; the reader can read and write the information of the RFID tag. The readers generally used are handheld and fixed. The fixed type can be divided into two types: channel type and small fixed type, as shown in Figure 1.

**Figure 2:**
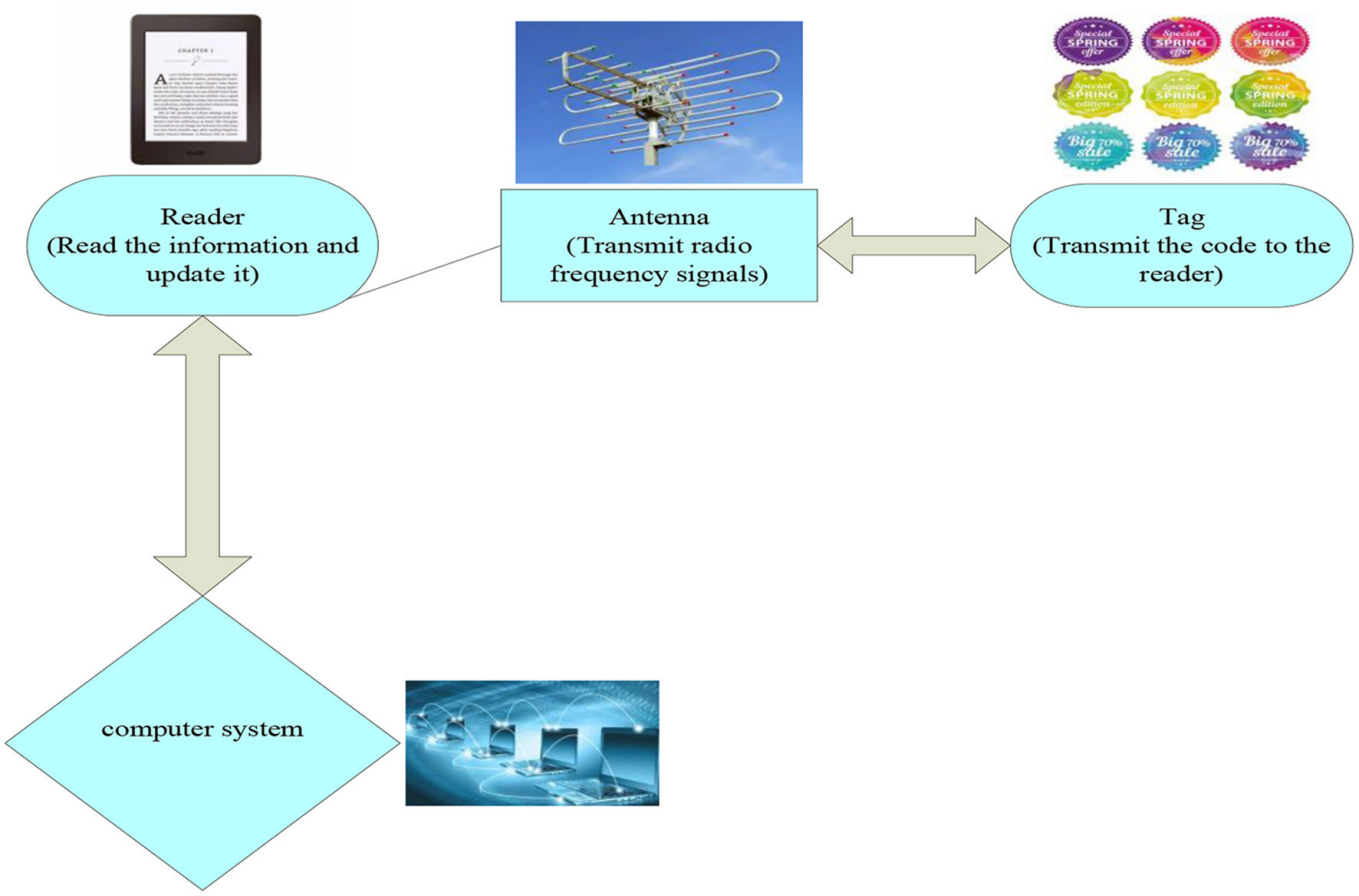
RFID working principle diagram.

The fundamental working theory of RFID is not complex. Its main elements are that the tag enters the magnetic field and sends a code to the reader, the antenna accepts the RF signal sent by the reader and uses the power gained by the sensing electrical field to send the message of the manufactured goods stored in the chip, or to activate a signal of a particular spectrum of frequencies. After the device reads and decodes the read information, the processed relevant data will be sent to the a central message system (16). Figure 2 shows the working principle of RFID technology.

According to various power modes of electronic tags, RFID can be divided into two categories: passive RFID and active RFID. There is no battery on the passive RFID electronic tag, and all the power required for work can be obtained by converting the electromagnetic wave sent from the received reader (17). The active RFID electronic tag is equipped with a battery that can use built-in power. Since the active area is formed around the tag, the remote radio frequency can also be identified by the reader. In the RFID system, the concept of the frequency band refers to the frequency range in which the reader transmits, receives and reads the tag signal through the antenna (18). From the perspective of application, the working frequency of the radio frequency tag is also the working frequency of the radio frequency identification system, which is one of the most important functions. The operating frequency of the RFID system not only affects the performance and size of the tag, affects the distance of the reader, but also affects the price of the tag and the reader, so the choice of frequency is very important (19). Table 1 mainly shows the specifications and characteristics of frequency bands used internationally. RFID tags are mainly divided into three categories: active, passive and semi-passive.

**Table 1:** RFID main frequency band standards and characteristics.

	**Low frequency**	**High frequency**		**UHF**	**Microwave**
Working frequency	125–134 KHz	13.56 MHz	JM13.56 MHz	868–915 MHz	2.45–5.8 GHz
Market share	74%	17%	2003	6%	3%
Reading distance	1.2 m	1.2 m	1.2 m	4 m	4 m
Speed	Slow	Medium	Soon	Quick	Soon
Humid environment	No effect	No effect	No effect	Greater impact	Greater impact
Directionality	Without	Without	Without	Part	Have
Applicable frequency	Yes	Yes	Yes	Part	Part
Existing ISO standards	11,784/85, 14,223	18,000- 3.1/14,443	18000-3.2/ 15,693,A,B,andC	EPC C0, C1,C2,G2	18000-4-
Scope of application	Natural gas	Library	Air transport	Trailer	Toll

From the above table, high-frequency RFID tags can more appropriately meet the needs of the food safety traceability system discussed in this article. If this frequency band tag is applied in fields such as bus cards, the stability is good and the price is relatively cheap. At the same time, high-frequency RFID tags have good storage properties, easy to read and write tags, and good security and reliability. In the end, high-frequency RFID was selected as the personal identification carrier in production.

#### Gps Technology

**Figure 3:**
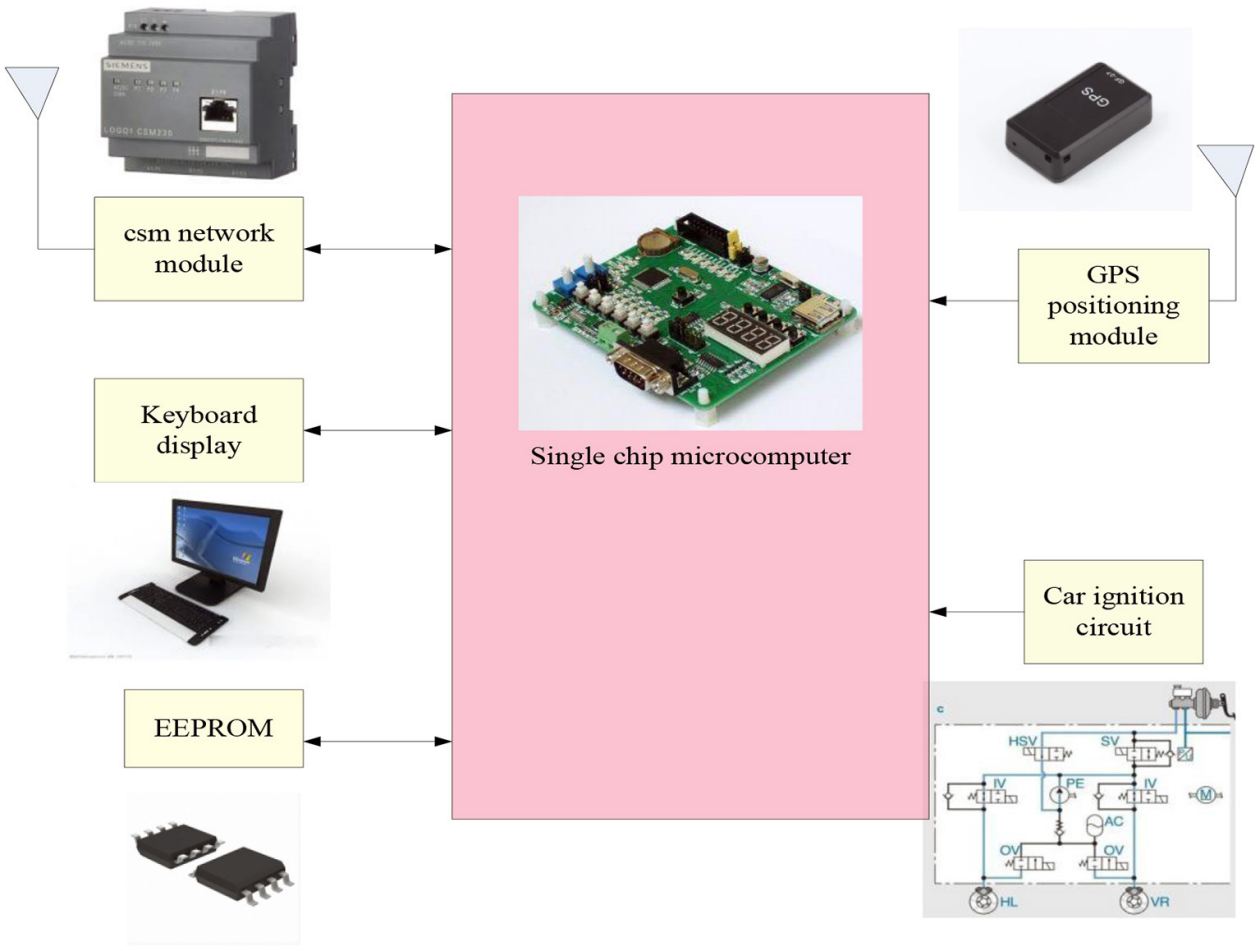
Flow chart of GPS positioning system.

GPS is a type of movement sensitive technique, which is an important technique to extend the network of things to mobile objects to collect information of mobile objects, and also an important technique for logistics intelligence and high level road traffic system (20). It is a high-precision radio navigation positioning system based on artificial earth satellites. It can provide accurate geographic location, vehicle speed and precise time information anywhere in the world and in near-Earth space. As a particularly important part of the food industry chain-modern logistics, GPS vehicle navigation system is a particularly important technology. With the use of GPS positioning technology and vehicle dynamic scheduling technology, logistics vehicles not only improve on-time operation, but also make it possible to find the route and stay time of transport vehicles during the entire transportation. This is to ensure the principle of food. The transparency of the entire transportation process of raw materials, finished products, and packaging materials provides a great guarantee (21). The working process of the car GPS positioning system is shown in Figure 3.

#### Video Surveillance System

The video surveillance system is also called closed-circuit television surveillance system (CCTV), as an important means to supervise the standards, safety and orderly work of the food production industry chain, and it also provides an important guarantee for the safety of food transportation (22). Monitoring is the physical basis for real-time monitoring of key departments or important places in various industries. Management departments can obtain effective data, images or sound information through it, and monitor and memorize the process of sudden abnormal events in time to provide efficient, Timely command and height, deploy police force, handle cases, etc. As an important part of various professional systems in food production, the video surveillance system is a professional communication method with certain security capabilities. It can pass through the front-end camera and its auxiliary equipment (codec, disk array, switch, display, etc.), and view the real-time monitoring images on site through a certain transmission method. At the same time, the video monitoring system can perform alarm linkage with fire protection, AFC and other systems, making the functions of the video monitoring system more complete and powerful. The video surveillance system transmits video signals in the transmission channel through transmission media such as optical fiber, coaxial cable, and network shielded cable. It adopts digital surveillance technology, video image compression technology, and digital network technology to form an independent and complete TV from the image display from the camera. The system, combined with the functions of multi-screen display, monitoring, compressed storage, retrieval and playback, and recording of digital video images, reflects the monitored objects in real time, vividly and truthfully, and records them through video monitoring storage devices, which can be widely used in various field of surveillance and security system (23).

**Figure 4:**
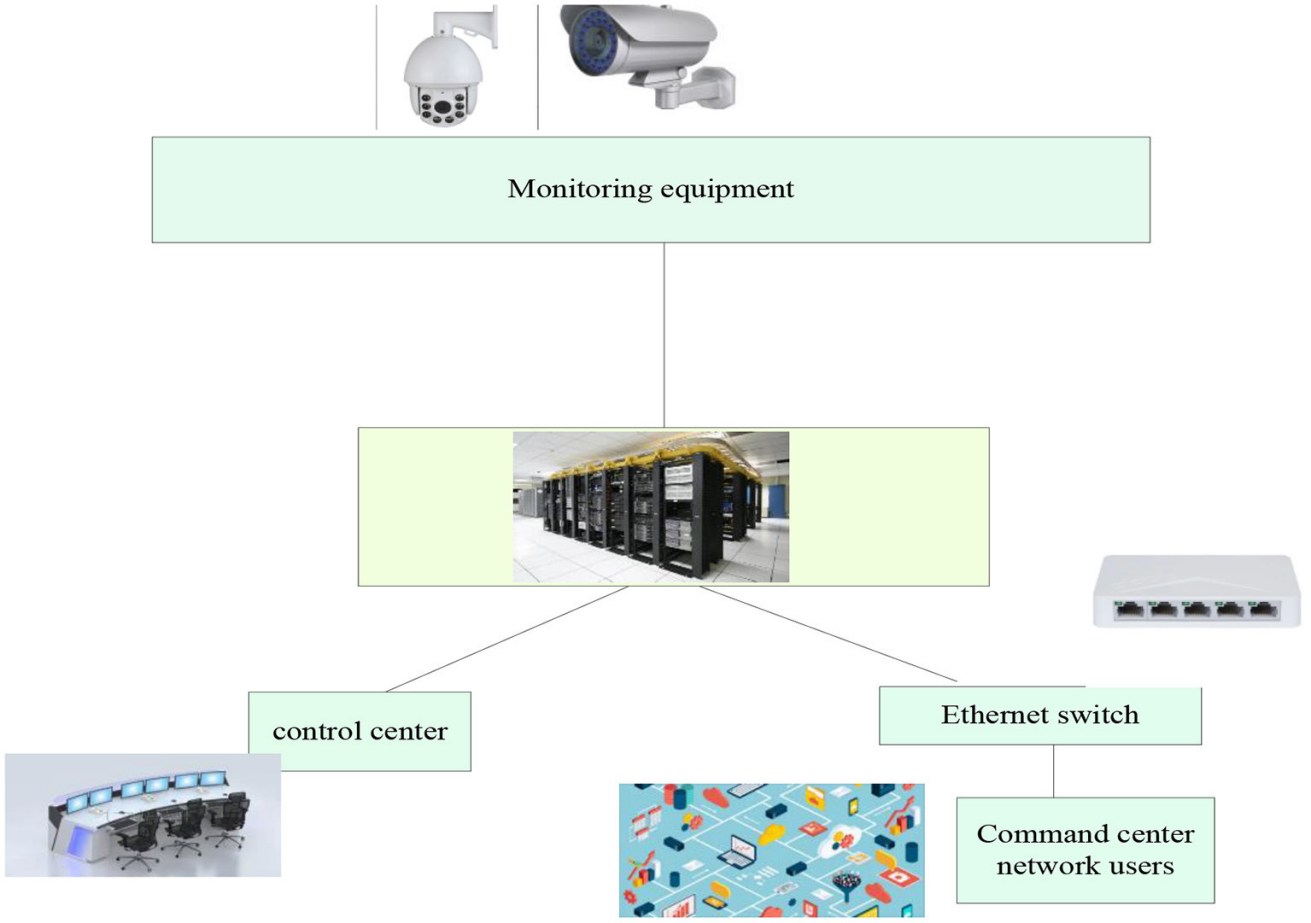
The basic structure of the video surveillance system.

The front-end equipment of the video surveillance system is usually composed of the camera body, lens (long focus/short focus), mounting bracket, protective cover, optical transceiver, optical cable splice box and other components. Each component is transmitted through a variety of transmission methods, such as coaxial cable, wireless AP or optical fiber transmission medium establishes a closed loop with the central switching equipment and server (24). In the actual video surveillance system, the front-end equipment will be selected according to the actual environment and conditions, but the camera and lens are the most basic components. Only the front-end has the video image collection, and the back-end video image storage and monitoring can be carried out. Figure 4 shows the basic structure of the video surveillance system.

### Image Sensor Technology

Sensors are a general term for devices that can sense prescribed measurement values and convert them into usable output signals according to specific rules (25). Some developed countries put sensor technology and communication technology in the same position. With the development of modern science, sensor technology is regarded as one of the three pillars of information technology. New fields closely related to modern science are also developing rapidly, among which are widely used. While promoting the development of various fields in the fields of industrial automation detection technology, aerospace technology, military engineering, medical diagnosis, etc.

#### Image Processing Related Technologies

##### Digital Image Processing

Digital image processing technology is a multi-disciplinary field. It uses computer to remove noise, enhance, restore, segment, extract features and other processing methods and technologies for images. Although its development history is not long, it has attracted extensive attention in various fields (26).

**Figure 5:**
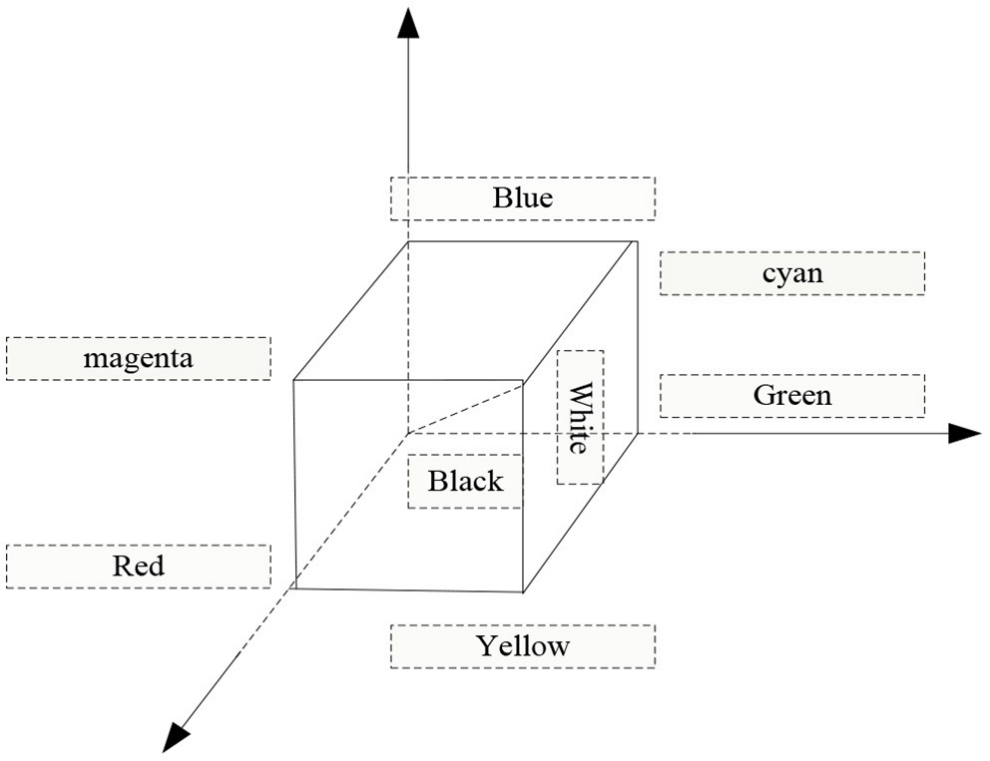
RGB color space.

The three colors of R, g, and b are the three primary colors of nature. The light with various color characteristics that people can usually see can be formed by mixing these three colors; on the contrary, the color characteristics of any kind of light can also be formed by r, g, b these three colors indicate. In the color space of the RGB model, three colors of r, g, and b can be used to establish a coordinate axis, and the values of these three colors are all assigned to 1, as shown in Figure 5.

This model is very common in our daily life, and most of the color images we usually see are based on this model. To make the color model more robust to illumination, the chrominance information and brightness information of the color must be separated from each other (27). As a result, people have developed several color models that separate chroma and brightness from each other. Such as YUV color model and HSV color model. YUV, is a color coding method. Often used in various video processing components. YUV allows for reduced bandwidth for chroma taking into account human perception when encoding photos or videos. In these models, Y is also the brightness of the color, while U represents the difference between red and green in the chromaticity of the color; V represents the difference between red and blue in the chromaticity of the color. The conversion relationship between YUV color model and HSV color model is as follows:

(1) $$\begin{array}{r} Y=0.299^{*}r+0.587^{*}g+0.144^{*}b \end{array}$$

(2) $$\begin{array}{r} U=-0.148^{*}r-0.289^{*}g+0.437^{*}b \end{array}$$

(3) $$\begin{array}{r} V=0.615^{*}r-0.515^{*}g-0.1^{*}b \end{array}$$

This model is obtained by separating the brightness and chrominance information of the color in the RGB model. The purpose of this is to compress the frequency band so as to have sufficient bandwidth (28).

The color space is somewhat different from the aforementioned YIQ and YUV color models. The color model uses the CCIR 601 coding scheme, y represents the brightness information of the color, cb represents the cyan information of the chromaticity, and cr represents the red information of the chromaticity. Because Cb and cr are independent of each other, they have the characteristics of two-dimensional plane clustering. This color model can also be converted from the RGB color model. The specific conversion formula is as follows:

(4) $$\begin{array}{r} \left[\begin{array}{c} y\\ cb\\ cr \end{array}\right]=\left[\begin{array}{c} 0\\ 128\\ 128 \end{array}\right]+\left[\begin{array}{c} 0.29900\\ -0.16874\\ 0.500000 \end{array}\begin{array}{c} 0.58700\\ -0.33126\\ -0.41869 \end{array}\begin{array}{c} 0.11400\\ 0.50000\\ 0.08131 \end{array}\right]\left[\begin{array}{c} r\\ g\\ b \end{array}\right] \end{array}$$

The chromatic model also takes into account the fact that the human eye is not sufficiently sensitive to chromatic changes in color, but is more responsive to the luminance of colors.

**Figure 6:**
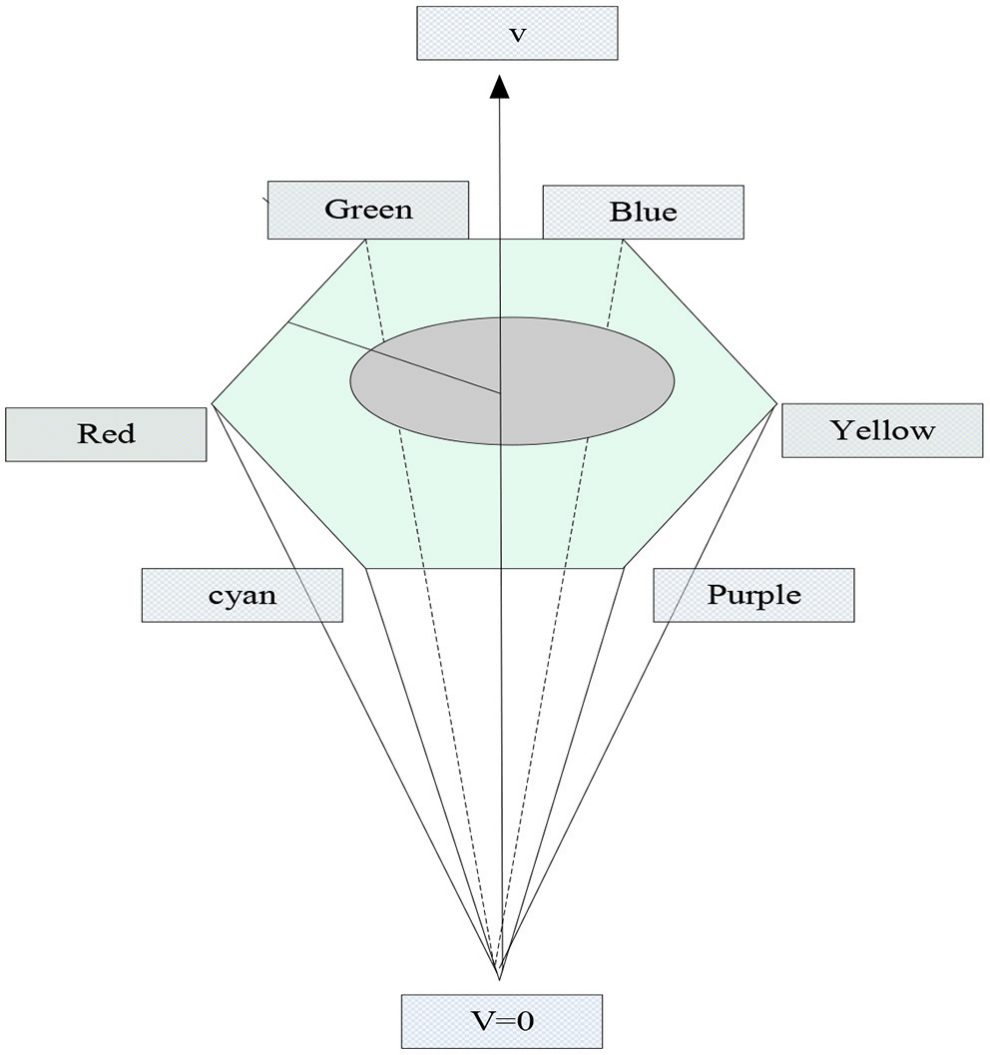
HSV color model.

HSV, which can also be called HSI, is a color model proposed by Munseu based on the separation of chroma, saturation, and brightness. HSV is an intuitive color model for the user. In this color model, H represents the chromaticity of the color information, that is, the chromaticity information of the color is represented by this one channel, unlike the previous color model, which is represented by multiple channels. S represents the saturation of the color information, that is, the depth of the color. V represents the brightness of the color information. The color model established in this way can be represented by a hexagonal pyramid, as shown in Figure 6.

The expression of the HSV color model can also be obtained through the conversion of the RGB color model. The obtained process is to first normalize the RGB color model, and then convert it to an HSV model. The conversion process is as follows: Normalization of RGB color model:

(5) $$\begin{array}{r} H^{\prime }=\{\begin{array}{c} \frac{e-t}{Max\left(q,e,t\right)-Min\left(q,e,t\right)}\\ \frac{t-q}{Max\left(q,e,t\right)-Min\left(q,e,t\right)}+2\\ \frac{q-t}{Max\left(q,e,t\right)-Min\left(q,e,t\right)}+4 \end{array}\begin{array}{c} \;q=Max\left(q,e,t\right)\\ \;e=Max\left(q,e,t\right)\\ \;t=Max\left(q,e,t\right) \end{array} \end{array}$$

Convert to HSV model:

(6) $$\begin{array}{r} H=\{\begin{array}{c} {H{}^{\prime }}^{*}60\\ {H{}^{\prime }}^{*}60+360\\ undefined \end{array}\begin{array}{c} \;H^{\prime }\geq 0\\ \;H^{\prime }<0\\ \;S=0 \end{array} \end{array}$$

(7) $$\begin{array}{r} S=\{\begin{array}{c} 0\\ 1-Min\left(q,e,t\right) \end{array}\begin{array}{c} V=0\\ V\neq 0 \end{array} \end{array}$$

(8) $$\begin{array}{r} V=Max\left(q,e,t\right) \end{array}$$

##### Maximum Between-Class Variance Method

This algorithm was researched and proposed by Japanese scholar Otsu Nobuyuki in 1979, it is an automatic threshold value method suitable for bimodal situations, also known as the Otsu method. This is an image segmentation method for segmenting grayscale images. It is named after its own name and is called the OTSU algorithm. Based on the characteristics of this algorithm, it can be named as the maximum inter-class covariance approach. Since this method performs segmentation of individuals according to the features of grayscale histogram, it is often classified as histogram segmentation in association with an issue of identity in image method. The idea of this method is to classify the image into two classes, one for the target class and the other for the background class. The task of the algorithm is to find the best segmentation threshold, which can make the image target class and background class the best separation. The steps for image segmentation using this algorithm are as follows:

If the total number of pixels of the entire image is set to W, then images with pixel gray levels of n share Wn, and the images always contain M gray levels, and we can get:

(9) $$\begin{array}{r} W=\overset{M-1}{\underset{n=0}{\sum }}W_{n}\left(n=0,1,2,\ldots ,M-1\right) \end{array}$$

Let the probability that a pixel whose grayscale is n is in the image is En. For En, there are:

(10) $$\begin{array}{r} E_{n}=W_{n}/W,E_{n}\geq 0,\overset{M-1}{\underset{n=0}{\sum }}E_{n}=1 \end{array}$$

Assuming that the segmentation threshold between the image target class and the background class is a, the target class is denoted as F1, and the background class is denoted as F2, we can get: (1) F1 contains all gray levels in (0, 1, 2, …, pixels between a); (2) F2 contains all pixels with gray levels between (a+1, a+2,…, n+1). Let Y be the probability of F1 and P be the probability of F2, then:

(11) $$\begin{array}{r} Y=\overset{n}{\underset{i=0}{\sum }}M_{n} \end{array}$$

(12) $$\begin{array}{r} P=\overset{n}{\underset{i=i+1}{\sum }}M_{n} \end{array}$$

Let A be the mean value of F1 and B be the mean value of F2, then:

(13) $$\begin{array}{r} A=\overset{n}{\underset{i=0}{\sum }}iM_{i}/Y \end{array}$$

(14) $$\begin{array}{r} B=\overset{n-1}{\underset{i=i+1}{\sum }}iM_{n}/P \end{array}$$

Let α1 be the variance of F1 and α2 be the variance of F2, then:

(15) $$\begin{array}{r} \alpha 1=\overset{n}{\underset{i=0}{\sum }}{\left(i-\mu_{0}\right)}^{2}M_{n}/Y \end{array}$$

(16) $$\begin{array}{r} \alpha 2=\overset{n-1}{\underset{i=i+1}{\sum }}{\left(i-\mu_{i}\right)}^{2}M_{n}/P \end{array}$$

Therefore, it is assumed that α1 is the intrastate covariance of objective and background classes F1 and F2, α2 is the intersubclass covariance of objective and background classes F1 and F2, and μ0 is the average gray level of the image. The following expressions can be obtained:

(17) $$\begin{array}{r} \alpha 1=Y\alpha 1+P\alpha 1=\overset{i}{\underset{i=1}{\sum }}{\left(i-\mu_{0}\right)}^{2}M_{n}+\overset{a-1}{\underset{i=i+1}{\sum }}{\left(i-\mu_{1}\right)}^{2}M_{n} \end{array}$$

(18) $$\begin{array}{r} \alpha 2=Y{\left(\mu_{0}-\mu_{1}\right)}^{2}+P{\left(\mu_{1}-\mu_{r}\right)}^{2}=YP{\left(\mu_{1}-\mu_{0}\right)}^{2} \end{array}$$

The maximum between-class variance method is more suitable for single-threshold segmentation. Artificial road signs with different colors are used in the article. When extracting, the artificial road signs need to be extracted one by one, which requires the extraction and distribution operation of each artificial road sign. For the extraction of artificial road signs of each color, it is a case of a single threshold segmentation, so the maximum between-class variance method is also more suitable for the image segmentation of this article.

### Food Safety Traceability System Structure

The so-called food safety traceability system is a targeted industry solution in the food safety system. My country has initially established some food traceability information systems and exchange platforms. These food safety traceability systems use RFID electronic tags, two-dimensional barcodes, wireless sensing and network transmission, video monitoring technology, etc., to automatically record the entire food supply chain information. And store the information in the corresponding data control system to realize data query and backtracking. Mainly refers to tracking the operation path of food raw materials according to the process of the supply chain, and the direction is from the producer to the consumer; while traceability refers to the reverse identification of the target food unit from the downstream consumer of the supply chain to the upstream producer, and through the record The scanning method traces back the supply process of a certain food monomer. Since the information of food production, processing, packaging, storage, transportation, sales and other links are recorded in the back-end database, consumers and supervisory departments can learn information about any link in the supply chain through electronic tags anytime and anywhere. As soon as a security incident occurs, determine the link where the problem occurred, define the responsible party, and take recall measures in a timely manner to minimize the loss of consumers and enterprises.

The traceability system is generally divided into five links, namely, production, packaging and processing, warehousing and transportation, sales, and supervision. This article mainly analyzes the supervision link.

Based on the hypothesis of “economic man,” one of the main pursuits of commercial enterprises is to maximize profits. As long as it is profitable, the enterprise will choose to take risks or even commit crimes. Therefore, the behavior of enterprises needs the supervision of government regulatory agencies. However, in real life because of this or because of that reason, the supervision work of the government supervision department is not so smooth, and food safety issues are still emerging. The following is an analysis of the game situation between the enterprise and the government supervision department from the perspective of game theory, and provides solutions for such problems.

The government supervision department has two choices of supervision and non-supervision for the production behavior of the enterprise. The enterprise also has two choices of producing qualified products and producing unqualified products. From the perspective of the government supervision department, it is assumed that the government supervision department adopts supervision. The expected benefits from non-regulatory measures are A1 and A2, respectively. According to some data, we can get:

(19) $$\begin{array}{r} A1=a\left(w-t\right)+\left(1-a\right)\left(w-t\right) \end{array}$$

(20) $$\begin{array}{r} A2=aw+{\left(1-a\right)}^{*}0 \end{array}$$

According to the solution method of the game Nash equilibrium strategy, let A1 = A2 to obtain

(21) $$\begin{array}{r} a=\left(w-t/w\right)=1-t/w \end{array}$$

According to the above formula, the probability n of an enterprise producing qualified products is related to the cost T and utility w obtained by the government supervision department in the supervision process. The smaller t or W is, the greater the probability a of an enterprise producing qualified products. Therefore, in order to ensure that an enterprise produces qualified products, the government supervision department should improve the supervision efficiency on the one hand. That is to reduce the supervision cost T. On the other hand, the higher authorities should affirm the work of the regulatory authorities and give appropriate awards, that is, increase effectiveness a.

## Food Traceability System Function Analysis and Database Design Experiment

### System Function Analysis

This topic is a food-based item traceability system, where the items are mainly agricultural products. My country's food safety supervision can be roughly divided into three stages: market access verification, production and sales supervision, and accident handling. The items are monitored from the soil, and the daily temperature and humidity, soil environment, etc. can be intuitively monitored. An environment with potential growth hazards raises alarms. After the goods are mature, they are marked with a unique QR code and sold on the e-commerce platform under the system. After purchasing this item, the user can scan it to query the entire growth, production, packaging, sales, circulation, production location, authenticity, etc. of the item.

The ultimate goal of food safety supervision is to ensure consumer safety, so that consumers can buy with peace of mind and eat with confidence. Therefore, we will establish an item traceability system for enterprise platform administrators to trace items. This system can also sell items online, providing consumers with purchases and also facilitating the follow-up tracking of items by enterprises. Therefore, this item traceability management system probably has the functions of item production source data supervision, corresponding item security QR code generation, item sales, logistics tracking, anti-counterfeiting traceability and other functions.

### Database Design

This system uses mysql database for development. Since mysql's innodb engine supports transactions, it can complete order payment and other functions. The structure of each table in the system database is as follows.

#### User Profile Table

It is used to record the basic messages such as user name, cell phone number and login password of registered users. The specific structure is shown in Table 2.

**Table 2:** User profile table.

**String name**	**Type of data**	**Field size**	**Primary key**	**Can it be empty**
ID	char	20	Yes	No
Name	varchar	32	No	No
Password	char	32	No	No
Sex	int	8	No	No
Paypasword	char	32	No	No
Phone	char	11	No	No
Address	varchar	20	No	No
Email	carchar	20	No	No
Accountid	int	8	No	No

#### Product Information Table

This table mainly records related information such as product number, name, inventory, price, shelf time, and corresponding QR code. The primary key of this table is the product ID, and the specific structure is shown in Table 3.

**Table 3:** Product profile table.

**String name**	**Type of data**	**Field size**	**Primary key**	**Can it be empty**
ID	char	20	Yes	No
Number	char	20	No	No
Name	varchar	32	No	No
Num	int	8	No	No
Price	float	11	No	No
Time	varchar		No	No
Type	int	8	No	No
Description	varchar	200	No	No
Path	varchar	20	No	No
Status	int	8	No	No
Sellerid	char	20	No	No
Codeid	char	42	No	No
Productid	int	11	No	No

#### Two-Dimensional Code Coding Table

This table mainly records the number of the two-dimensional code and its usage status. 0 means not activated, 1 means activated, and 2 means destroyed. The specific structure is shown in Table 4.

**Table 4:** Two-dimensional code coding table.

**Field name**	**Type of data**	**Field size**	**Primary key**	**Can it be empty**
ID	char	42	Yes	No
Status	int	11	No	No
Code	char	32	No	No

#### Verification Record Table

This table mainly records the corresponding QR code number, scanning time, scanning location, scanner's mobile phone model, scanning times, last scanning time, etc. The specific structure is shown in Table 5.

**Table 5:** Verification record table.

**Field name**	**Type of data**	**Field size**	**Primary key**	**Can it be empty**
ID	char	42	Yes	No
Time	int	11	No	No
Lon	float	17	No	No
Lat	float	17	No	No
TypeId	int	11	No	No
Num	int	11	No	No
Lastscantime	int	11	No	No

## Analysis of Related Research and Experiment Results of Food Traceability System

### Experimental Sample Analysis

A total of 1,691 people participated in this experiment, including 580 men and 1,111 women. It can be concluded from the gender that women are significantly more concerned about food safety than men. From the analysis of age distribution, the proportion of middle-aged people participating in the survey is relatively high, followed by adolescents' attention to food safety, young consumers have a high tolerance for quality and safety, and children and elderly people's attention to food safety is relatively low. However, this survey needs to state that there are many elderly people who are willing to participate in the survey. However, a considerable number of residents in this age group have certain shortcomings in writing, so some elderly people did not fill in this survey form.

### Analysis of Survey Results

**Figure 7:**
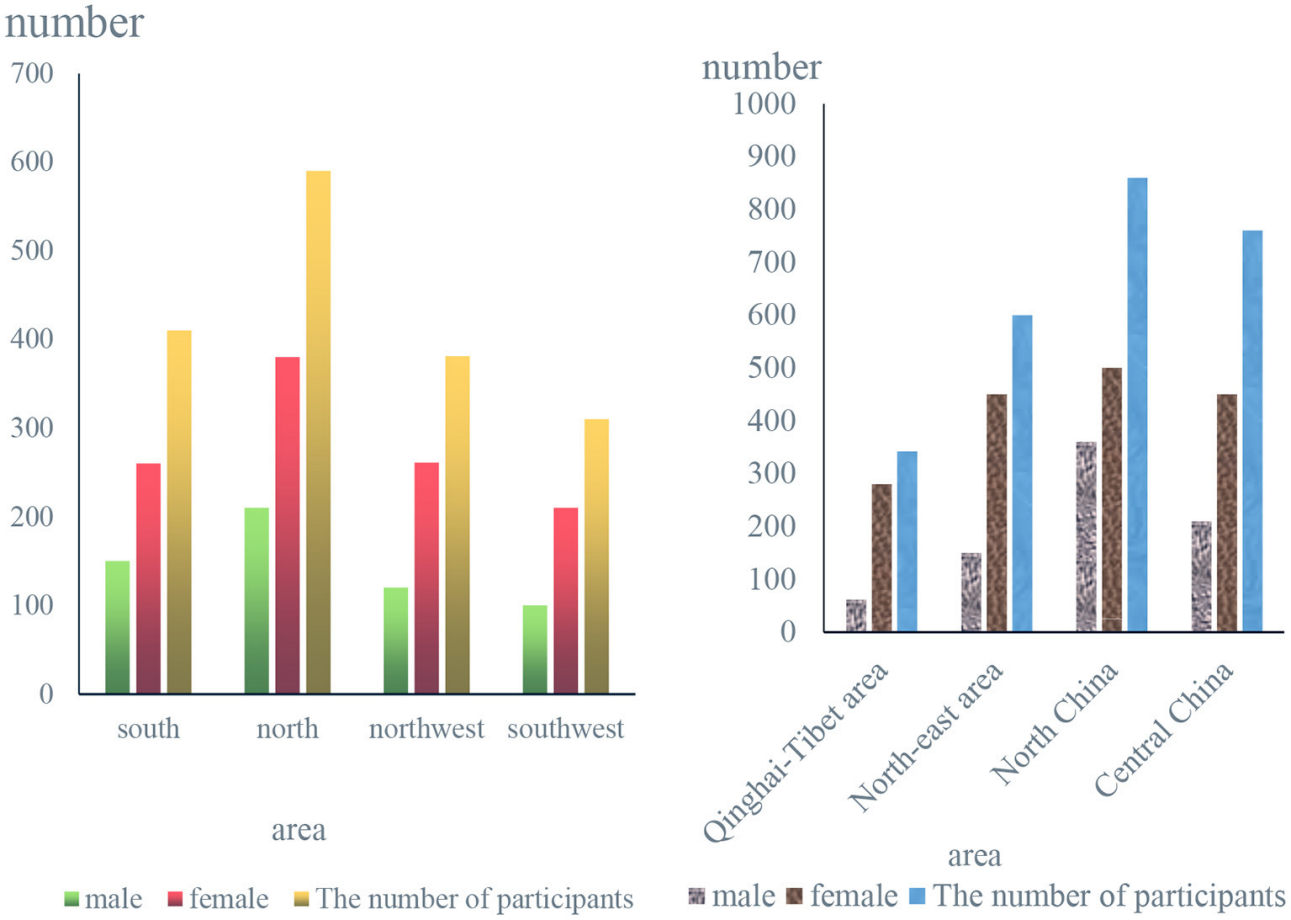
Number of men and women concerned about food safety by region.

According to Figure 7 and the previous survey results of this topic, the statistical results can be analyzed, and a comparison chart of male and female residents' concerns about food safety in various regions can be obtained, as shown in Figure 7.

From Figure 7, we can conclude that regardless of the number of people in that region, the number of women concerned about food safety is often higher than 40% of that of men. According to the survey data in the figure, the economies of South China and North China are more economical. People in developed areas also pay more attention to food safety.

**Figure 8:**
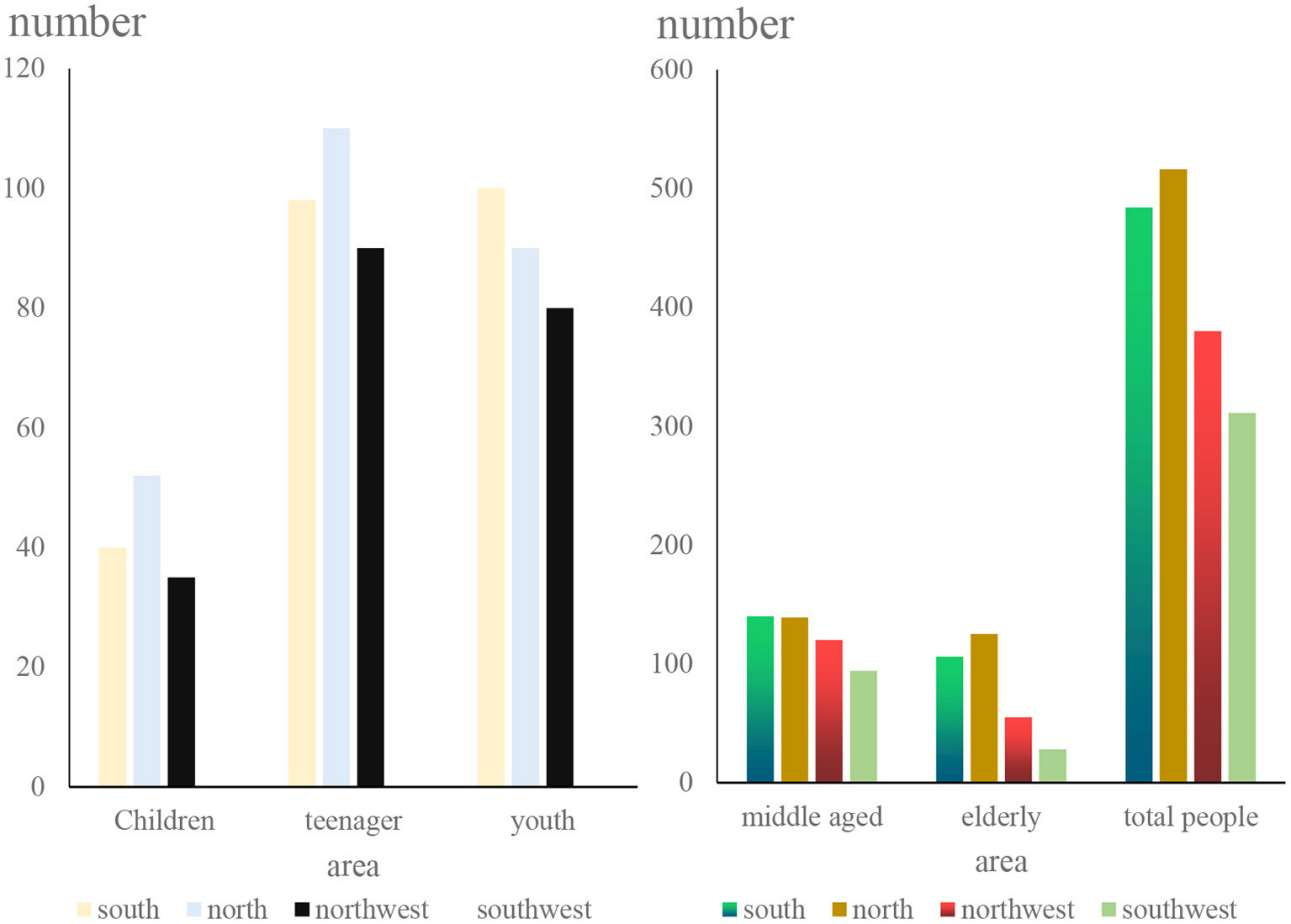
Statistics of the level of attention paid to food safety by age groups.

In addition to conducting research and analysis on people of different genders, we also compiled a comparison chart of people of different ages on food safety concerns, as shown in Figure 8.

From Figure 8 we can conclude that middle-aged people pay the most attention to food safety, followed by teenagers and young people. It can also be seen from the figure that in this survey process, more than 30% of the personnel came from the northern region, the Southwest is the least and may be affected by regional economic and cultural factors, which can show that in this field survey, the northerners paid the most attention to food safety.

**Figure 9:**
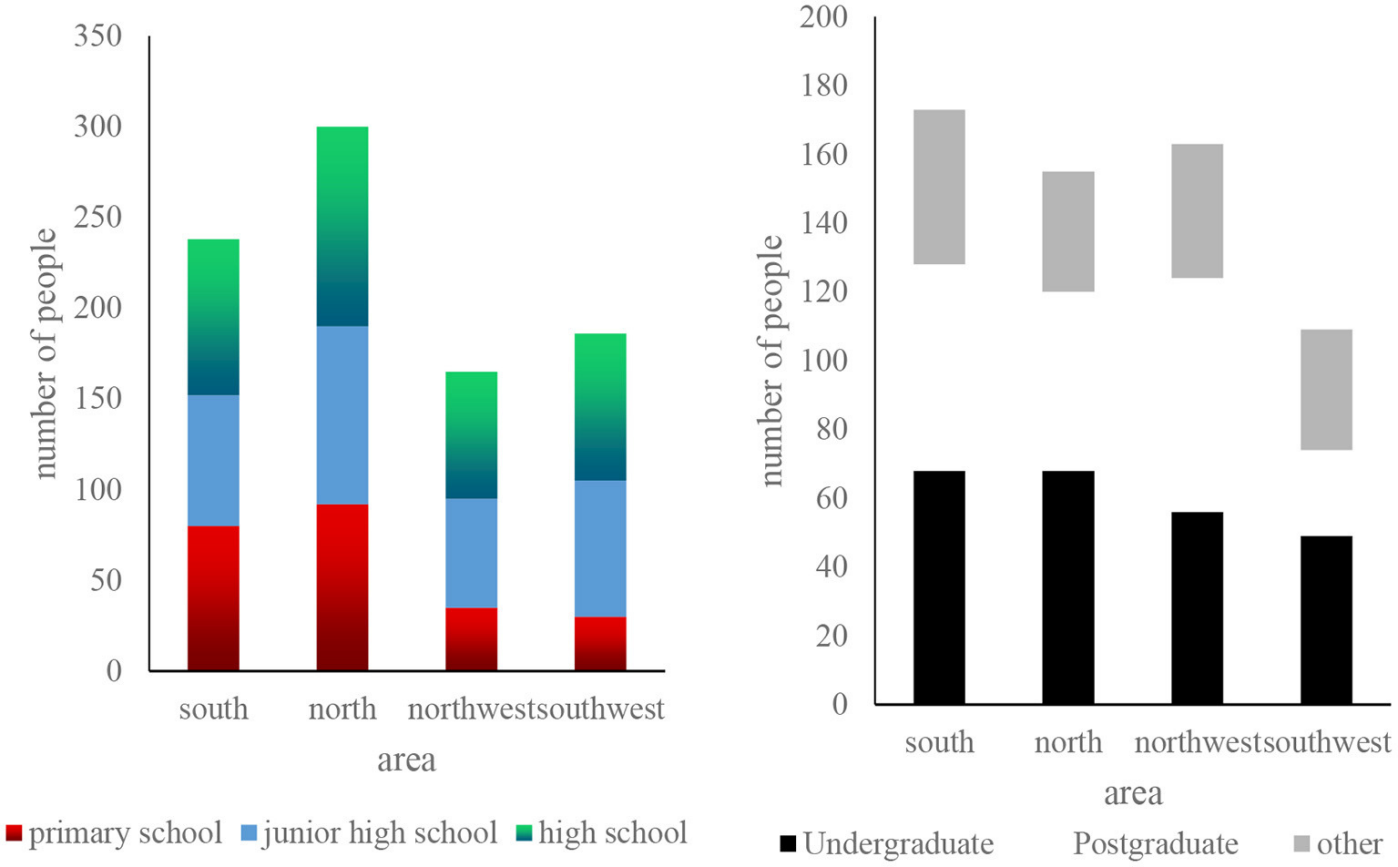
Statistical table of the survey personnel's educational background distribution.

In the statistics of this and other survey results tables, we also organized and summarized people with different educational backgrounds. Figure 9 shows the specific statistics table we made.

From Figure 9 we can know that people with middle or high education accounted for more than 35% of the total number of our survey, indicating that people with higher education are more concerned about food safety, but there are still most of them with junior high school education. There is also a high degree of concern for existing food safety.

**Figure 10:**
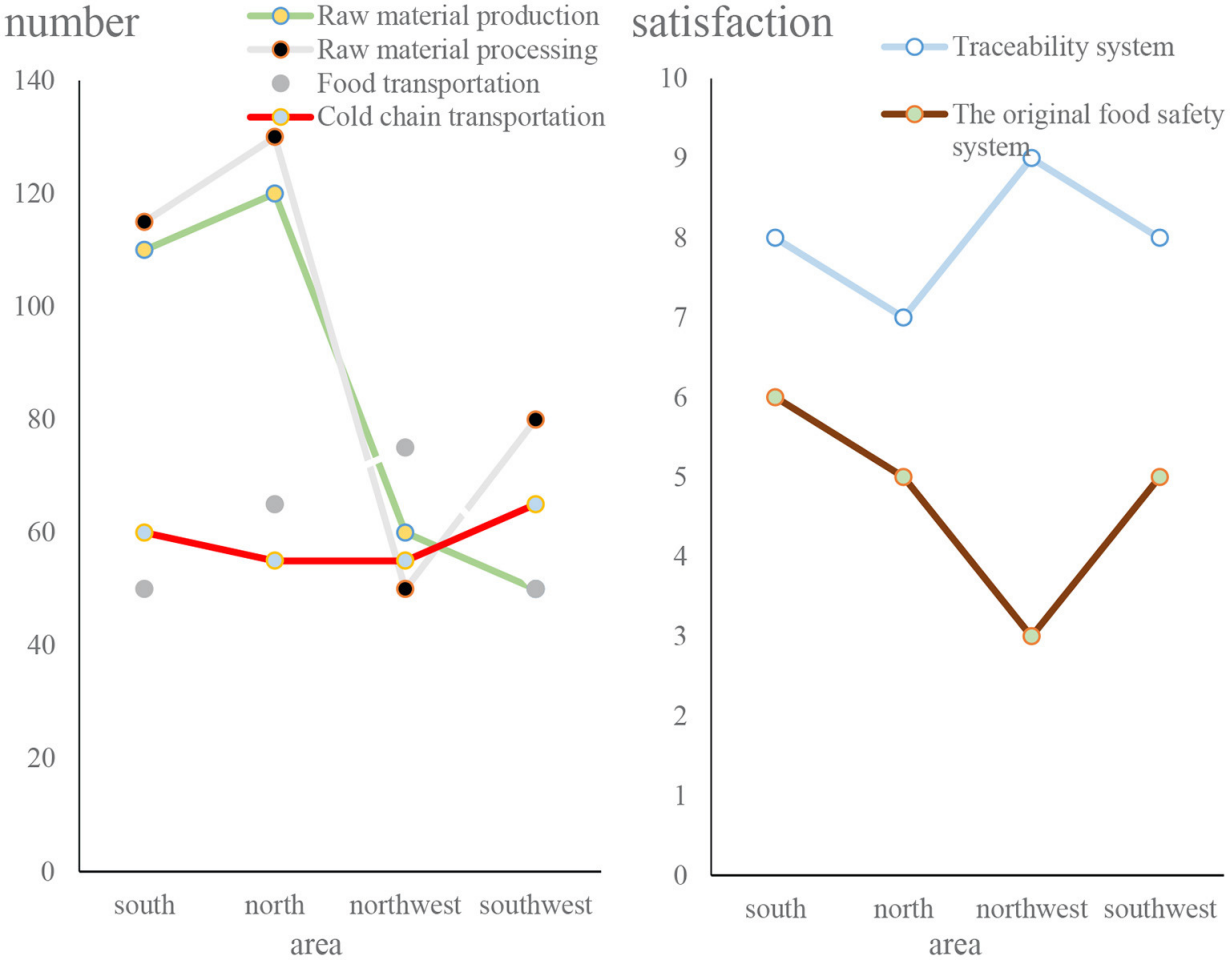
Figure of the results of investigations on the food production process and traceability system.

We then obtained some data and made relevant statistics from the investigation of food safety production links and traceability system. The specific situation is shown in Figure 10.

From Figure 10, we can conclude that in the entire food production process, more than 36.5% of the people pay more attention to food processing and the production of food raw materials, and in the food safety system, people are satisfied with the food safety traceability system. The degree is 15.6% higher than the original food safety system.

## Conclusions

The establishment of a food information safety traceability system based on the Internet of Things technology is a long-term and complex project, which is the basis for realizing food safety informatization. This paper studies the food information safety traceability system based on the Internet of Things technology. First, it conducts a detailed study on the Internet of Things technology and the food traceability system, and then conducts a survey on people's food safety related content. The survey results show that there are more than 40% of women are more concerned about food safety, and people are more concerned about food raw materials and processing. By using the Internet of Things technology to create a food safety information system, not only can the production quality of the food produced in the production process be improved, and the entire food can be transparent in the production, processing, and transportation of raw materials, but also make people aware of the entire food system and the food industry chain. The security information traceability system is more satisfactory.

## Data Availability Statement

The original contributions presented in the study are included in the article/supplementary material, further inquiries can be directed to the corresponding author.

## Ethics Statement

Ethical approval for this study and written informed consent from the participants of the study were not required in accordance with local legislation and national guidelines.

## Author Contributions

SL: write. QS: analysis. NZ: check. All authors contributed to the article and approved the submitted version.

## Funding

This work was supported by Social Science Foundation Major Projects of China (18ZDA102) 2018 and Youth Project of Natural Science Foundation of China (72004089) 2020.

## Conflict of Interest

The authors declare that the research was conducted in the absence of any commercial or financial relationships that could be construed as a potential conflict of interest.

## Publisher's Note

All claims expressed in this article are solely those of the authors and do not necessarily represent those of their affiliated organizations, or those of the publisher, the editors and the reviewers. Any product that may be evaluated in this article, or claim that may be made by its manufacturer, is not guaranteed or endorsed by the publisher.
